# New Insight into Metal Ion-Driven Catalysis of Nucleic Acids by Influenza PA-Nter

**DOI:** 10.1371/journal.pone.0156972

**Published:** 2016-06-14

**Authors:** Daria Kotlarek, Remigiusz Worch

**Affiliations:** Laboratory of Biological Physics, Institute of Physics, Polish Academy of Sciences, Warsaw, Poland; Hong Kong University of Science and Technology, HONG KONG

## Abstract

PA subunit of influenza RNA-dependent RNA polymerase deserves constantly increasing attention due to its essential role in influenza life cycle. N-terminal domain of PA (PA-Nter) harbors endonuclease activity, which is indispensable in viral transcription and replication. Interestingly, existing literature reports on *in vitro* ion preferences of the enzyme are contradictory. Some show PA-Nter activity exclusively with Mn^2+^, whereas others report Mg^2+^ as a natural cofactor. To clarify it, we performed a series of experiments with varied ion concentrations and substrate type. We observed cleavage in the presence of both ions, with a slight preference for manganese, however PA-Nter activity highly depended on the amount of residual, co-purified ions. Furthermore, to quantify cleavage reaction rate, we applied fluorescence cross-correlation spectroscopy (FCCS), providing highly sensitive and real-time monitoring of single molecules. Using nanomolar ssDNA in the regime of enzyme excess, we estimated the maximum reaction rate at 0.81± 0.38 and 1.38± 0.34 nM/min for Mg^2+^ and Mn^2+^, respectively. However, our calculations of PA-Nter ion occupancy, based on thermodynamic data, suggest Mg^2+^ to be a canonical metal in PA-Nter processing of RNA *in vivo*. Presented studies constitute a step toward better understanding of PA-Nter ion-dependent activity, which will possibly contribute to new successful inhibitor design in the future.

## Introduction

Influenza has caused outbreaks every 1–3 years repeatedly throughout past 400 years [1], however the effectiveness of current methods of treatment is still doubtful [2–4]. According to WHO, the annual number of sufferers amounts to 3–5 million severe cases and 250–500 thousand deaths worldwide. Adaptive mutations and genetic reassortment are the main reasons of high intraspecific variability, increase in virulence and drug resistance [5–8].

Influenza RNA-dependent RNA polymerase (RdRP) is a heterotrimeric complex of one acidic subunit (PA) and two basic ones (PB1 and PB2) [9, 10]. PA harbors endonuclease activity [11–13], which is employed in cap-snatching mechanism by cleaving 10–13 nucleotide fragments from 5’ end of host capped pre-mRNA bound by PB2. These RNA oligonucleotides are eventually utilized as primers in viral mRNA synthesis by PB1 [14, 15]. PA subunit, truncated to its ~209 N-terminal domain (PA-Nter), has full endonucleolytic activity and its fold was shown to be similar to those of type II restriction endonucleases, involving the binding of bivalent metal ions Mn^2+^ [11] or Mg^2+^ [13]. PA-Nter deserves constantly increasing attention [16, 17] and is considered as a target for inhibitors, although the details of metal-dependent catalysis are not fully characterized.

PA-Nter cleaves single stranded RNA and DNA [18] accordingly to two-metal-ion mechanism [19], similarly to all identified polymerases and self-splicing ribozymes [20–23]. This mechanism implies that the first ion, located at site M1, supports formation of attacking nucleophile by withdrawing electrons, while the second at site M2 facilitates the exit of leaving group through neutralization of its negative charge. Both ions stabilize the transition state [21, 24, 25]. However, intensive research brings a contradiction in number and nature of ions on both biochemical and structural aspects. S1 Table demonstrates only the most essential discrepancies of PA-Nter ion dependence described below in some more details.

Using 81-nt panhandle RNA as a substrate and 1.5 mM divalent metal ions, strong activity of PA-Nter with Mn^2+^ and weak activity with Mg^2+^ at pH 8 was observed [11]. However, at pH 7 cleavage was observed only in the presence of Mn^2+^ and Co^2+^. In contrast, PA-Nter activity on U-rich 51-nt RNA in the presence of either 1 mM Mg^2+^ or Mn^2+^ was reported [26]. For 20-nt RNA, exclusive cleavage with 1mM Mn^2+^ was reported [27]. Similarly, cleavage of 7.249 kb ssDNA plasmid with 1mM Mg^2+^ was not observed but only with 1mM Mn^2+^ [28]. However, 15-nt molecular beacon were cleaved with either 1mM Mg^2+^ or Mn^2+^. The results on the endonuclease activity using viral ribonucleoprotein and full PA are also puzzling. Doan *et al*. [19], who used the same substrate as Datta *et al*., estimated the maximum activity for RNP with either 100 μM Mn^2+^ or 100 μM Co^2+^ and 2-fold lower activity with 1 mM Mg^2+^. Interestingly, Noble *et al*. [29] detected the strongest full PA activity with Mn^2+^, but still strong with Mg^2+^, Co^2+^ and Zn^2+^. Furthermore, PA-Nter structures do not fully clarify the results of biochemical studies.

Structural reports are not consistent concerning the type and number of ions [11, 13, 30, 31], presenting one Mg^2+^ in site M2 [13] or two Mn^2+^ in M1 and M2 [11, 30] in the structures of PA-Nter. Yuan et al. [13] showed that Mg^2+^ ion occupies site M2 and is directly coordinated by Glu80, Asp108 and three water molecules stabilized by His41, Glu119, Leu106 and Pro107. The site M1 in the Yuan`s structure is occupied by water molecule coordinated by H41. The structure of Dias et al. [11] contains two Mn^2+^ atoms in the active site. The first atom occupies site M1 and is coordinated by Asp108, Glu119, His41, Ile120 and residue Glu59 from a neighboring monomer. However, the contribution of residue Glu59 is questioned by Liu et al. [17] and Xiao et al. [32] who attributed its involvement to crystallization artifact. The second Mn2+ ion was localized in site M2 and it is coordinated by Glu80, Asp108 and two water molecules. The residue Asp108 serves as a bridge between two metals. In the study of Kowalinski et al. [31] ions are coordinated by His41, Asp108, Glu119, Ile120 and two water molecules in M1 or Glu80, Asp108 and four water molecules in M2. Moreover, in the presence of Mg alone, this study showed no metal in M1 and Mg^2+^ bound to M2. DuBois et al. [30] localized two Mn^2+^ ions in both sites coordinated by the same residues as in [31] Recent structures of complete polymerases showed two Mg^2+^ ions coordinated in the PA subunit in the complex from Influenza C [33] or Influenza B viruses [34]. However, from the above description, it is clear that for a more detailed analysis of metal-dependent cleavage, a wider set of experimental conditions must be exploited.

Here, to elucidate contradictory PA-Nter ion-dependent activity, we complemented the results of gel electrophoresis with fluorescence cross-correlation spectroscopy (FCCS), a technique allowing for monitoring the reaction in real time with single molecule sensitivity. Our experiments demonstrate a significant impact of the co-purified ions on PA-Nter activity and calculations show the importance of Mg^2+^ in catalysis in cells. Carefulness in interpretation of the results carried out for differently purified proteins *in vitro* and the difference in ion preference *in vivo* is noteworthy to consider in successful inhibitor design.

## Materials and Methods

### Chemicals

Fluorescently labelled 20-nt RNA (5’ Cy5-GAA UAC UCA AGC UAU GCA UC-3’ fluorescein) used previously by [27] and unlabeled 30-nt DNA hairpin (5’ GGG GG A_20_ CC CCC 3’) were custom-synthesized by FutureSynthesis (Poland). 30-nt DNA hairpin of the same sequence labeled with 5’ Atto 488 and 3’ Atto 647N (PAGE quality) as well other oligonucleotides: 19-nt DNA (5’ ATG GCT AAT GAC CGA CAG C 3’), 66-nt DNA (5' ATG GCT AAT GAC CGA CAG CTG GGA TCC GAA TTC AAT ATT GGT ACC TAC AAG CTT TGC GCT CGT ATC 3') (HPLC quality) were from IBA (Germany). M13mp18 plasmid (7.249 kb) was purchased from New England Biolabs (USA). MgCl_2_, MnCl_2_, DTT, 40% 29:1 acrylamide/bis solution and TBE buffer were purchased from Sigma Aldrich. Alexa Fluor 488 and Alexa Fluor 647 fluorescent dyes for microscope confocal volume calibration were from Life Technologies.

### PA-Nter

N-terminal domain of PA subunit (strain A/USA:Huston/AA/1945 H1N1) was delivered by MyBioSource (USA) as a stock solution of 6.3 μM. The His-tag protein was overexpressed in *E*. *coli* and purified by nickel affinity chromatography, followed by ion-exchange chromatography, to guarantee the purity higher than 90% (by SDS-PAGE, S1 Fig). The stock solution was kept in 20 mM Tris-HCl, 0.5 M NaCl, pH 8, 50% glycerol. Prior to experiments, the stock buffer was exchanged using Illustra NAP-5 column (GE Healthcare Life Sciences) according to provided protocol. 250 μl of stock PA-Nter was applied onto column, eluted with 1 ml of buffer (50 mM Hepes and 150 mM KCl pH 7.8) and used further in cleavage studies. Protein-rich fractions contained ~50% of the stock concentration as verified with Micro BCA Protein Assay Kit (ThermoFisher).

### Electrophoresis

Urea polyacrylamide gel (urea PAGE) and agarose electrophoresis (AE) assays were performed to test the endonucleolytic reaction in the micromolar regime of substrate. 20-nt RNA or 19-nt, 30-nt and 66-nt DNA were incubated with PA-Nter in the presence of 0.25–1 mM Mg^2+^ or Mn^2+^ at 25°C for 1 h. Concentrations of reagents are specified in figure legends. Next, samples were denatured by adding 1 μl of 40 mM EDTA in formamide and incubation at 70°C for 5 min. Control time course cleavage was done by stopping the reaction in 10 min steps. Urea polyacrylamide gel (20%) was pre-run in TBE buffer (200V) for 30 min. Subsequently, samples were loaded and run for 2 h. M13mp18 plasmid was incubated with PA-Nter in the presence of 0.25–1 mM Mg^2+^ or Mn^2+^ at 37°C for 2 h and inactivated in 80°C for 20 min. Samples were loaded onto 1% agarose gel and run in TAE buffer (90 V) for 45 min. Gels were stained with ethidium bromide and distained with distilled water for 15 min, respectively, next trans-illuminated by ChemiDoc™ MP System (Bio-Rad) and analysed in ImageJ (http://imagej.nih.gov/ij/). Fluorescent images of gels showing the cleavage of 30-nt fluorescent DNA hairpin were registered with Typhoon Trio imager with default settings (GE Healthcare).

### Confocal setup and FCCS measurement

All FCCS measurements were performed on Zeiss LSM 780 confocal microscope system equipped with ConfoCor 3 unit. Samples were excited by two beams of an Ar-ion (488 nm) and a He-Ne (633 nm) lasers. The excitation beams passed through a water-immersion objective (C-Apochromat 40x/1.2 NA) forming in the sample confocal volume element in the femtoliter range. Emission collected with the same objective was separated from the excitation light by main dichroic beam splitter (MBS 488/561/633) and focused onto a 35 μm pinhole. Signals were split into green and red channels by using second dichroic mirror (NFT 635 vis) and filters (BP 495–555 and LP 655, respectively) and were registered by avalanche photo diodes. Confocal volumes with lateral radii *ω*_*0*,*g*_ and *ω*_*0*,*r*_ were calibrated every time prior measurements by using fluorescent dyes Alexa 488 and Alexa 647 using known diffusion coefficients (414 and 300 μm^-2^s^-1^, respectively). To investigate the enzyme kinetics, different concentrations of PA-Nter were added to ~15 nM 30-nt DNA hairpin solution in 50 mM Hepes and 150 mM KCl (pH 7.8). Enzyme dilutions were freshly prepared before each measurement. All reactions were carried out in a droplet of 40 μl on cover glass (Carl Roth, #1.5 high precision). Reaction kinetics was monitored for about 30 min (30 runs x 30 s, point to point time 60 s). During this time sample evaporation was negligible.

For the analysis we applied home-written script in Python performing automatic fitting routine for each time point in the output Zeiss fcs file. Auto- and cross-correlation curves were evaluated applying a standard one component 3D diffusion model including a triplet term using:

$$G_{i}(\tau )={G(0)}_{i}{\left(1+\frac{T_{i}}{1-T_{i}}exp(-\frac{\tau }{\tau_{t,i}}))(1+\frac{\tau }{\tau_{d,i}}\right)}^{-1}{\left(1+\frac{\tau }{S^{2}\tau_{d,i}}\right)}^{-\frac{1}{2}}$$ (1)

The triplet fraction was kept zero for cross-correlation curve. Amplitudes were corrected for background and cross-talk between channels (with *κ* = 0.015 as determined in separate measurement for the same laser power) according to [35]. Maximum cross-correlation *cc*_*max*_ achieved in the setup, calculated as *cc*_*max*_ *= min(G(0)*_*g*_,*G(0)*_*r*_*)/G(0)*_*cc*_ for the 30-nt DNA hairpin was checked to be almost identical with the one obtained for doubly labeled dsDNA 66-nt fragment (79.5 ± 3.2%). The concentration of doubly labeled *C*_*gr*_ species monitored during reaction was calculated as:

$$C_{gr}=\frac{{G(0)}_{cc}}{{G(0)}_{g}{G(0)}_{r}V_{eff,cc}}$$ (2)

with the effective cross-correlation volume *V*_*eff*,*cc*_ *= Sπ*^*3/2*^*ω*_*0*,*eff*_^*3*^ with a lateral radius *ω*_*0*,*eff*_ *= ((ω*_*0*,*g*_^*2*^*+ω*_*0*,*r*_^*2*^*)/2)*^*1/2*^ and a structural parameter S = 5.

### smFRET

smFRET pulse interleaved excitation (PIE) experiments were carried out on Zeiss LSM 710 microscope equipped with PicoQuant LSM upgrade kit for time-resolved measurements. As an excitation source two LHD lasers with emission wavelengths of 485 nm and 640 nm were used. The confocal volume was maintained by using water (C-Apochromat 40x/1.2 NA) objective and focusing the emitted light onto a 35 μm pinhole. Donor and acceptor signals were separated from each other by using a second dichroic mirror (545nm) and filters: 520/35 bandpass and 635 longpass, respectively and further recorded by two avalanche photodiodes. For checks of the 30-nt DNA hairpin conformation and PA-Nter processivity, ~40 μl droplet was placed in home-made chambers consisting of cut 0.5 ml Eppendorf tubes glued (Norland Optical Adhesive 63) to a glass cover slip (#1.5, high precision Carl Roth) sealed with parafilm. The measurement was done for an hour in the same buffer as for FCCS, supplemented with 1mM Trolox. The histogram analysis was done using SymPhoTime ver 5.3.2.3 with an implemented PIE_FRET_2D_burst_analysis_05 script (PicoQuant).

### Binding calculations

In the discussion we used the following equations describing PA-Nter ion occupancy. We based on the isothermal calorimetric (ITC) data [26] providing equilibrium binding constants (*K*_*M1*_ and *K*_*M2*_) for both M1 and M2 sites for Mg^2+^ and Mn^2+^. Assuming non-cooperative binding, the average number of ions bound to PA-Nter *ν* is given by

$$\nu (x,K_{M1},K_{M2})=\frac{x}{K_{M1}+x}+\frac{x}{K_{M2}+x}$$ (3)

where *x* denotes molar concentration of ion.

For the discussion regarding metal ion occupancy of PA-Nter in living cells, we used a dependence of protein-ion complex [*P•I]* on the total protein concentration *P*_*tot*_ and total ion concentration *I*_*tot*_ bound to protein with an equilibrium constant *K*_*d*_. It is obtained as a root of a quadratic equation describing the equilibrium of free ion *I*_*f*_ and protein *P*_*f*_ and *P•I* complex formed (*P*_*f*_ +*I*_*f*_ ⇔ *P•I*).

$$[P∙I]=1/2∙\left\lceil I_{tot}+K_{D}+P_{tot}+{\left({\left(I_{tot}+K_{D}\right)}^{2}+{\left(P_{tot}+K_{D}\right)}^{2}-{2K}_{D}^{2}-2I_{tot}P_{tot}\right)}^{1/2}\right\rceil$$ (4)

## Results

### Ion-dependent PA-Nter cleavage of substrates varied by length

**Fig 1:**
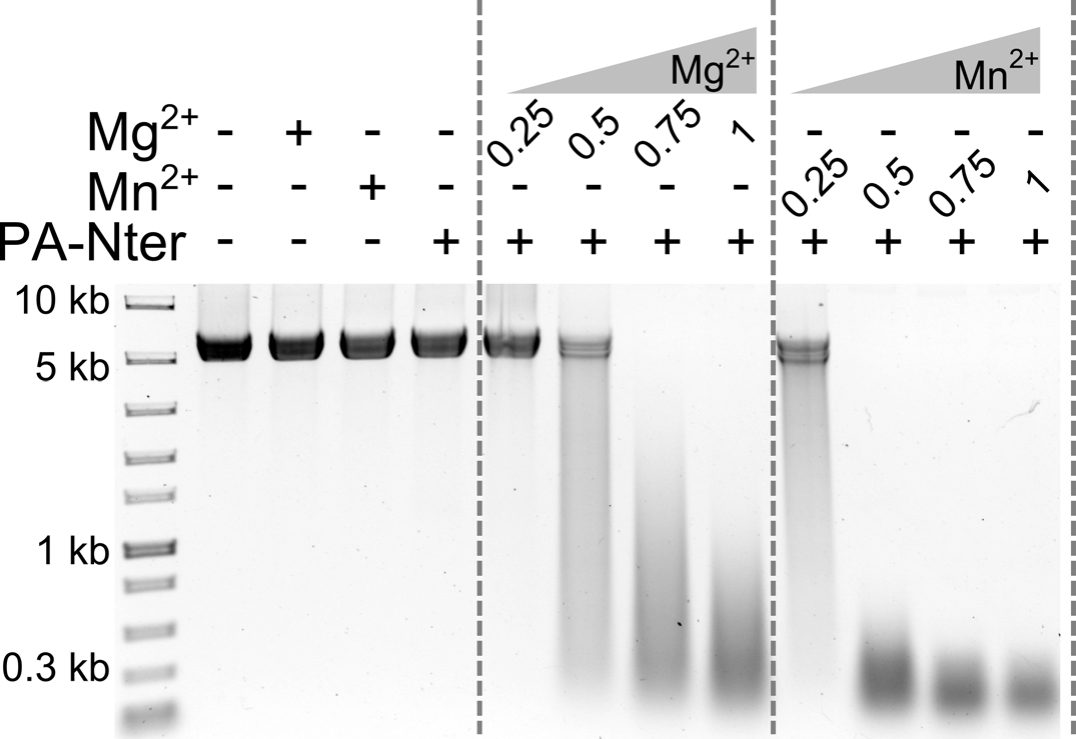
Cleavage of ssDNA plasmid depends on ion concentration. Products of the cleavage of ssDNA M13mp18 plasmid by PA-Nter in the presence of increasing concentrations of divalent metal ions separated by agarose gel electrophoresis. All samples were incubated at 37°C for 2 h and inactivated at 80°C for 20 min (reaction of 500 ng M13mp18 with 1.25 μM PA-Nter).

To quantify PA-Nter ion-dependent activity, we first performed the reaction on ssDNA plasmid M13mp18 with an increasing concentration of Mg^2+^ or Mn^2+^, varying from 0.25 to 1 mM. As we expected, we observed no cleavage in the control samples, indicating that plasmid did not degrade in the presence of Mg^2+^ or Mn^2+^, neither during the incubation at 37°C and endonuclease required metal ions for its activity (Fig 1). Enzymatic reaction of PA-Nter on ssDNA M13mp18 plasmid resulted in an increased activity of enzyme accordingly to the growing concentration of either Mg^2+^ or Mn^2+^. These results point out that PA-Nter is active in the presence of both ions, but its performance is strictly dependent on the type and concentration of metal. Under the applied experimental conditions, the measurable activity of PA-Nter was already observed in the presence 0.25 mM Mn^2+^, while minimally 0.5 mM Mg^2+^ was necessary for the cleavage catalysis (Fig 1). However, this was no longer true for PA-Nter purified with no extra buffer exchange step (S2 Fig). To highlight the expected differences we increased enzyme/substrate molar ratio by taking half of the plasmid and twice of the protein. In this case, even the control sample without addition of ions was efficiently cleaved, indicating the content of divalent ions in the stock solution. Under such conditions, growing concentration of added Mg^2+^ or Mn^2+^ did not fully reveal the metal dependent enzymatic activity. This points to the impact of co-purified ions in the measurements of PA-Nter activity.

**Fig 2:**
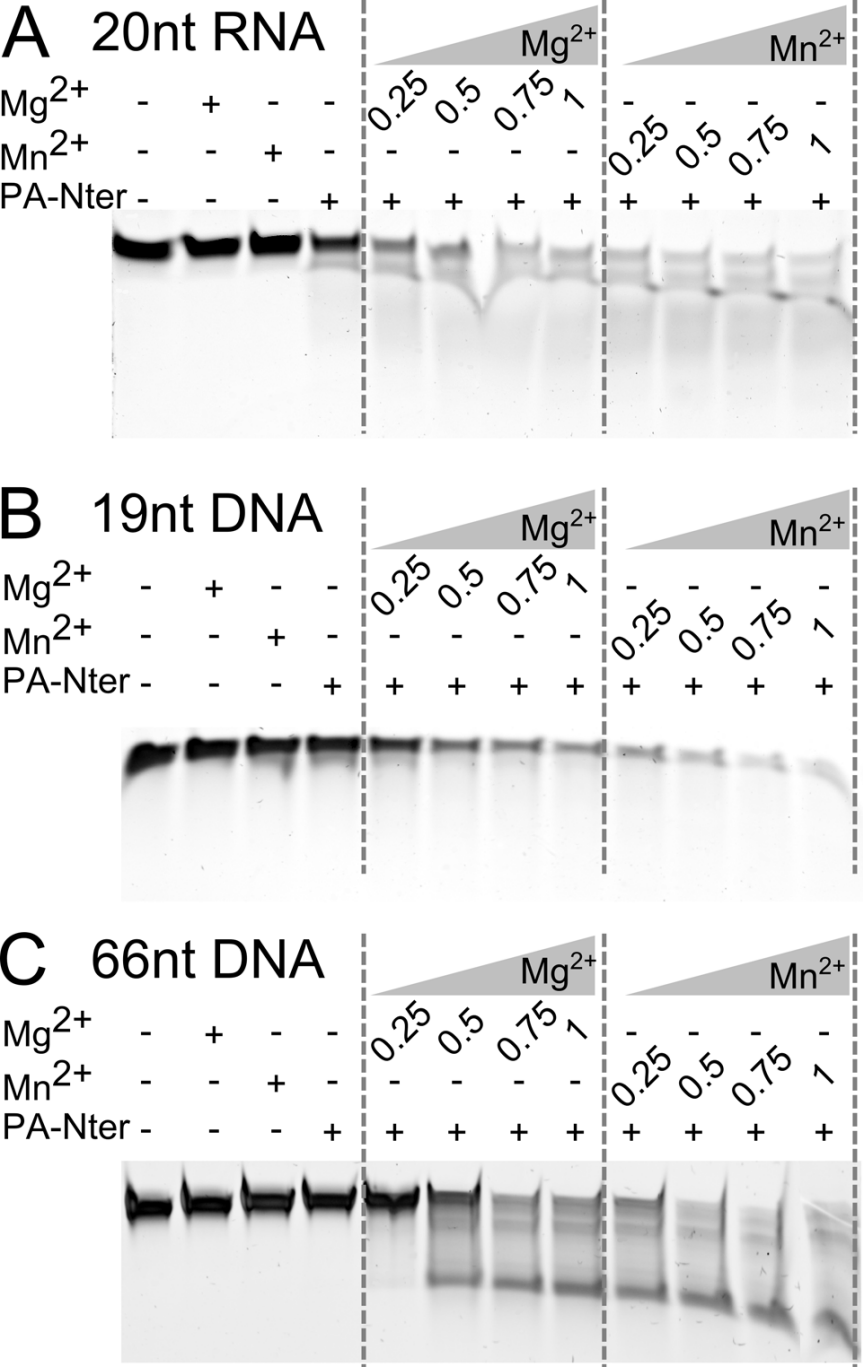
Cleavage of short nucleic acids fragments. Activity of PA-Nter on the various nucleic acids substrates in the presence of increasing concentrations of divalent metal ions. All samples were incubated with purified 2.55 μM PA-Nter at 25°C for 1 h and inactivated at 70°C for 5 min. (A) 6.8 μM 20-nt RNA, (B) 8.5 μM 19-nt DNA, (C) 2.4 μM 66-nt DNA. Possible secondary structures of these oligonucleotides are shown in S3 Fig.

To further examine the activity of PA-Nter on different substrates in the presence of increasing concentrations of divalent metal ions, we performed the reaction with purified endonuclease using 20-nt RNA, 19-nt DNA and 66-nt DNA (Fig 2). Consistently with experiment in Fig 1, we used the same control samples. Fig 2 shows PA-Nter activity on all substrates used in the presence of both ions, while growing metal concentration accelerated the reaction. Similarly to Fig 1, PA-Nter exhibited slightly higher activity in the presence of Mn^2+^ than Mg^2+^. For short substrates (20-nt RNA and 19-nt DNA) we observed a slight effect of partial cleavage without addition of metal ions (Fig 2A and 2B). Interestingly, in corresponding control sample cleavage did not occur when longer 66-nt DNA substrate was used (Fig 2C), although its molar concentration was the lowest. In spite of our experimental effort, further purifications of the enzyme by dialysis and/or one extra ion-exchange chromatography (also preceded by EDTA chelation) did not lead to lowering the content of putative bound-to-protein ions (data not shown). Thus the impact of co-purified ions on PA-Nter activity is further confirmed.

### Single molecule fluorescence-based assays for cleavage characterization

**Fig 3:**
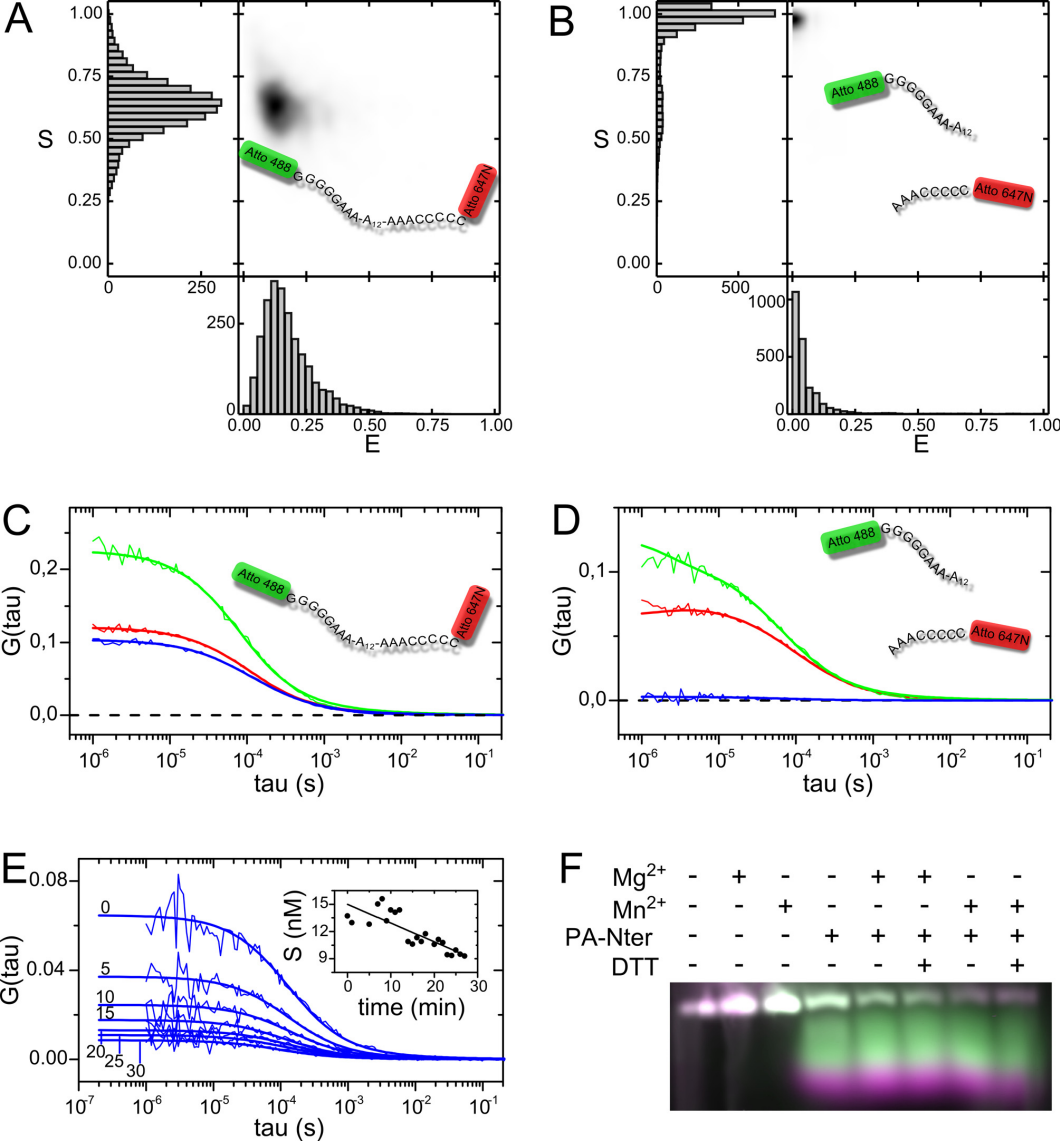
Single molecule fluorescence techniques applied for PA-Nter activity measurements. Histograms of single molecule FRET efficiency of 30-nt fluorescent hairpin DNA (A) before the reaction, (B) after the reaction with PA-Nter; E = 0 indicates no FRET, E = 1 indicates maximal FRET, S denotes photon stoichiometry. Example histogram shown from a series of three independent experiments. The correlation functions of hairpin DNA measured with FCCS, (C) maximum cross-correlation before the reaction, (D) minimum cross-correlation after the reaction with PA-Nter. Blue line indicates cross-correlation, green and red denote auto-correlation functions. (E) Cross-correlation functions in 0, 5, 10, 15, 20, 25 and 30 minute of the reaction of 15 nM hairpin DNA with 100 nM PA-Nter. Insert picture shows the concentration of the hairpin DNA (nM) throughout the reaction time. (F) Comparison with electrophoretic assay. Products of the cleavage of 5.4 μM hairpin DNA with 3.3 μM PA-Nter in the presence of 1 mM ions and 2 mM DTT. Samples were incubated at 25°C for 1 h and inactivated at 70°C for 5 min.

Next, we developed single molecule fluorescence-based assays to most accurately characterize cleavage catalysis of PA-Nter in the presence of either Mg^2+^ or Mn^2+^. For this purpose, we used 30-nt hairpin DNA terminally labelled with fluorescent dyes Atto 488 and Atto 647N. First, we performed single-molecule FRET (smFRET) analysis of hairpin DNA applying pulsed interleaved excitation (PIE) scheme to assess the conformation population in the buffering conditions used for cleavage. PIE is beneficial since it allows for differentiation of FRET-competent molecules (containing donor and acceptor) from molecules lacking one of the fluorescent dyes. Fig 3A and 3B demonstrate FRET histograms, in which photon stoichiometry (S) was plotted versus FRET efficiency (E). Here E = 1 denotes high efficiency of FRET, indicating population of molecules having closed conformation, while E = 0 means reverse. Photon stoichiometry indicates the ratio of molecules bearing donor and acceptor, and thus S = 0 denotes molecules lacking donor, S = 0.5 indicates equal number of donors and acceptors per molecule, while S = 1 describes population of molecules without acceptor [36]. Prior to the catalysis, 30-nt hairpin DNA exhibited low FRET efficiency with peak in E = 0.11 and photon stoichiometry in S = 0.63, pointing that open conformation of hairpin is prevalent and the sample did not contain detectable donor only-labelled species (Fig 3A). However, after the cleavage the position of peak changed to E ~ 0 and S ~1, indicating no more intact molecules in the sample and thus confirming PA-Nter activity (Fig 3B).

Further we performed a series of fluorescence cross-correlation spectroscopy (FCCS) experiments using the same DNA hairpin as a substrate. This technique also exhibits single molecule sensitivity and first was used for investigation of enzymatic reaction by Kettling *et al*. [37]. Doubly labelled molecules with spatially and spectrally separated fluorescent dyes are exposed to the two laser beams during their passage throughout the confocal volume. Emitted fluorescence signals from red, green and red-green molecules are registered and further proceeded into auto- and cross-correlation curves, respectively. Each auto-correlation amplitude (G(0)_r_, G(0)_g_) is formed from emission signals of particular dye attached to mono- and doubly labelled species. However, cross-correlation (G(0)_cc_) derives exclusively from doubly labelled molecules, as for 30-nt DNA hairpin (Fig 3C). By monitoring of G(0)_cc_, it is possible to assess if the reaction produces or consumes doubly labelled species and hence, the concentration of substrate or product in each reaction stage (Eq 2). Cleavage of DNA hairpin resulted in G(0)_cc_~0 (Fig 3D). Similarly as in electrophoretic experiments, in which reaction kinetics could be observed by stopping the reaction after several time points (S4 Fig), we first performed control experiments to check if cleavage occurs in the absence of added metal ions. Under the conditions used for FCCS, we did not observe such behavior, neither an unspecific degradation of substrate (S5 Fig). Since the hairpin DNA substrate was fluorescent, it was also possible to show the cleavage on a fluorescent image of the electrophoretic gel, where after cleavage separate bands for each of the fluorophores could be resolved (Fig 3F). Both bands were rather smeared, however the 3’ terminal Atto647N-containing part migrated faster. Electrophoretic experiment performed on an unlabeled substrate gave similar result, indicating that the fluorescent labels did not influence the enzyme processivity (S6 Fig).

**Fig 4:**
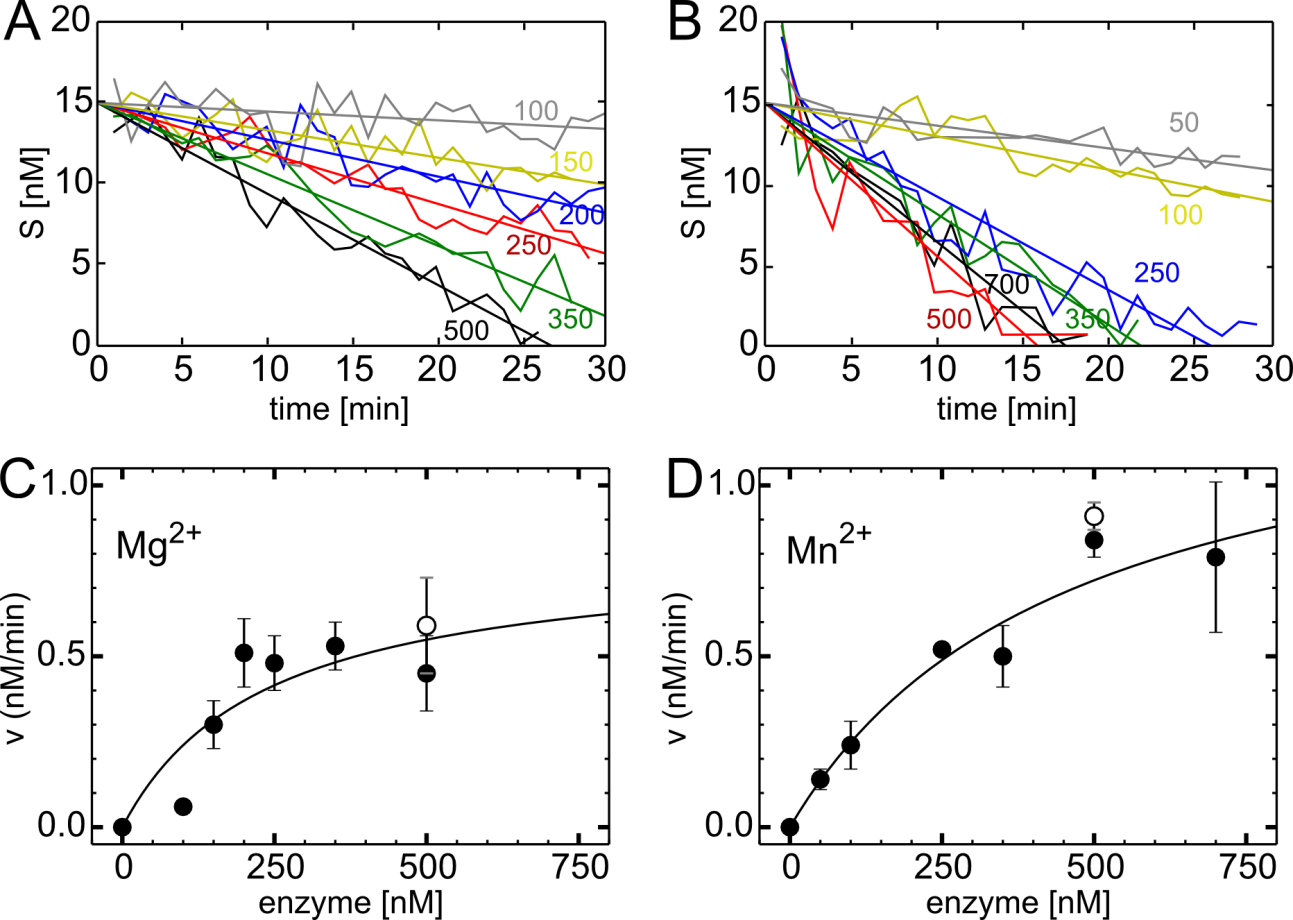
Cleavage kinetics. Example data set showing the decrease of substrate concentration in time in for a range of PA-Nter concentrations (numbers in [nM]) in the presence of A) Mg^2+^, B) Mn^2+^. (C, D) Reaction rate of PA-Nter in the presence of either 1 mM Mg^2+^ or 1 mM Mn^2+^ in the function of enzyme concentration. The kinetics was monitored in the regime of enzyme excess and constant concentration of doubly labelled hairpin DNA (~15 nM) using fluorescence cross-correlation spectroscopy (FCCS) for 30 min. Errors are depicted as SEM from triplicate measurements. The line shows fitted hyperbolic dependence: (*v*_*max*_ *• x)/(K*_*M*_ *+ x)*. The fitting parameters for asymptotic maximum reaction speed *v*_*max*_ were 0.81± 0.38 and 1.38± 0.34 nM/min for 1 mM Mg^2+^ and 1 mM Mn^2+^, respectively. Black points indicate the measurement in the reaction buffer containing 50 mM Hepes, 150 mM KCl pH 7.8 and 1 mM ions, white points (not included in fitting) indicate the measurement in the reaction buffer containing 50 mM Hepes, 150 mM KCl pH 7.8, 2 mM DTT and 1 mM ions.

By using Eq 2 we determined the initial concentration of substrate c = 18.3 ± 5.7 nM (average from all starting points, *n*>100), what was consistent with the prepared concentration (15 nM). During the reaction course, we observed constantly decreasing amplitude of cross-correlation curve, what directly implicates the consumption of doubly labelled species of hairpin DNA by the endonuclease (Fig 3E). The inset picture represents an example of substrate concentration plotted versus time of reaction allowing for determination of reaction velocity from the slope, which was further used to obtain kinetics of reaction in the presence of Mn^2+^ and Mg^2+^ (Fig 4A and 4B).

### Kinetics of PA-Nter monitored by fluorescence cross-correlation spectroscopy

Having validated the FCCS technique for monitoring of DNA hairpin cleavage, we quantified the kinetics of catalysis in presence of Mg^2+^ and Mn^2+^ ions. Low processivity of PA-Nter forced us to perform these experiments in the excess of enzyme over the substrate, since in the typical experiments for Michaelis-Menten *v*_*max*_ and *K*_*M*_ determination we did not observe cleavage of nanomolar substrate by a less concentrated enzyme. Therefore we kept the concentration of DNA hairpin constant to 15nM and added different amounts of PA-Nter. Under such conditions, a concentration dependent reaction speed could be resolved (Fig 4). We observed a linear dependence of substrate concentration for each of the enzyme concentration (Fig 4A and 4B), allowing us to determine the reaction velocity from the curve slope. For averaged values we fitted a hyperbolic equation to obtain the asymptotic maximum reaction speed *v*_*max*_, which was 0.81± 0.38 and 1.38± 0.34 nM/min in the presence of 1 mM Mg^2+^ and 1 mM Mn^2+^, respectively (t-test p-value 0.31). In agreement with electrophoretic experiments, we observed PA-Nter activity for both of metal ions, with a ~1.7-times better performance of Mn^2+^.

### Impact of reducing conditions

Noting that some authors have used reducing agents in the reaction buffer for PA-Nter catalysis, we investigated if they have any significant influence on the PA-Nter activity. Fig 3F present products of the cleavage catalysis of 30-nt fluorescent hairpin DNA in the presence of Mg^2+^ or Mn^2+^ and 2 mM DTT, since such concentration of DTT was used in the reaction buffer by Datta *et al*. [27]. Comparable activity of PA-Nter with both ions and no measurable impact of 2 mM DTT on the PA-Nter performance was observed. We checked also the impact of DTT in FCCS experiments for one of the higher enzyme concentration used. Here again, within the uncertainties, the difference of reaction speed in presence of 2mM DTT was negligible (Fig 4). Moreover, we investigated the catalysis in buffer consisted of 50 mM Tris-HCl, 100 mM NaCl 10mM β-mercaptoethanol pH 8 (S7 Fig), since this reducing agent was previously used by Stevaert *et al*. [28]. We proceed with the cleavage catalysis on ssDNA plasmid M13mp18, maintaining the concentration of PA-Nter, substrate and ions the same as in Fig 1. The results of both assays were identical, confirming that reducing agents did not change the PA-Nter activity nor the cleavage pattern.

## Discussion

Facing growing pharmaceutical relevance of PA-Nter and emerging inconsistency concerning type and number of divalent metals involved in the enzymatic reaction, we investigated PA-Nter activity in the range of Mg^2+^ or Mn^2+^ concentrations. Contrary to other authors who did not report PA-Nter activity with Mg^2+^ [11, 27] or reported partial activity under particular conditions (Dias et al. 2009) or substrates [28], we observed the catalysis in the presence of both ions Mg^2+^ either Mn^2+^, irrespectively of buffer and substrate used. In agreement with other studies [19, 29], we observed slightly higher activity with Mn^2+^. Our study highlights metal concentration-dependent regulation of the enzyme, while the impact of co-purified ion and enzyme/substrate molar ratios used in particular experimental approach should be also taken into consideration. Furthermore, we confirm the reducing agents do not change the activity of endonuclease.

### Enzyme processivity

The reported here *v*_*max*_ values at the order of 1 nM/min may appear to be slow. However, in the values as small as 0.06–0.09 nM/min were reported for Dicer-2 enzyme (Ribonuclease III class) [38] or ~0.6•10^−3^ nM/min for human RNase H1 [39]. These enzymes have nanomolar Michaelis *K*_*M*_ constant, resulting in their processivity at the level of 10^5^−10^8^ M^-1^s^-1^. In the case of PA-Nter, micromolar *K*_*M*_ were reported: 2.4 μM for 20-nt RNA [27] and 0.23 μM for 15-nt molecular beacon [28], indicating that its efficiency may be lower. Although a direct determination of *K*_*M*_ is not possible from the presented here way of kinetics measurements, measurements in the regime of small substrate concentrations ([S]<< *K*_*M*_), allow for a determination of *k*_*cat*_*/K*_*M*_ value. This is because:

$$V_{0}=\frac{V_{max}}{1+K_{M}/[S]}≅\frac{V_{max}}{\frac{K_{M}}{\left[S\right]}}=\frac{k_{cat}}{K_{M}}[E_{0}][S]$$ (5)

From the linear regime of PA-Nter kinetics measured here, we can estimate *k*_*cat*_*/K*_*M*_ at the level of ~2•10^3^ M^-1^s^-1^, similar for both metall ions. Indeed this value is smaller than the mentioned above values for other nucleic acid-cleaving enzymes. Probably the relatively low processivity of PA-Nter is increased when the enzyme works together with PB2 unit binding effectively the 5’ mRNA cap of the cleaved mRNA chain.

### Metal-dependent regulation of PA-Nter

**Fig 5:**
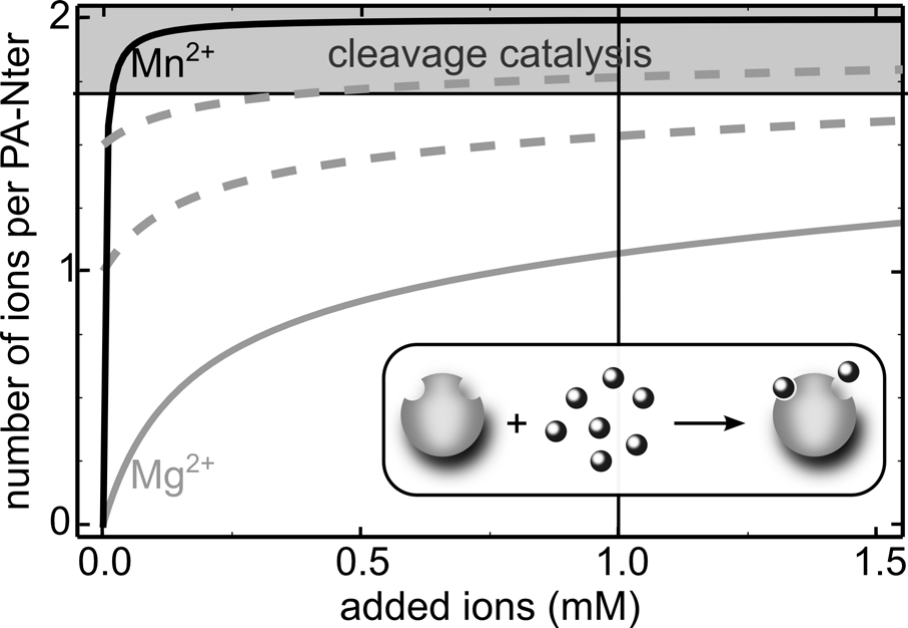
PA-Nter saturation with metal ions accordingly to binding constants. The enzymatic catalysis of PA-Nter is dependent on the number of ions occupying the active site of the enzyme.

Two-ion mechanism was proposed as a fingerprint for all DNA and RNA polymerases and self-splicing ribozymes [20–23, 25]. Accordingly, PA-Nter being a metallonuclease belonging to PD-(D/E)XK family, was suggested to follow this model [19]. Here we propose a putative explanation for the lack of PA-Nter activity in the presence of Mg^2+^ reported by some authors, basing on the thermodynamic data on ion binding by PA-Nter [26]. Fig 5 shows the average number of ions per PA-Nter molecule as a function of added ions, assuming independent binding of ions to M1 and M2 sites (see Materials and Methods for details). Owing to its 500-fold higher affinity to PA-Nter [26], Mn^2+^ quickly saturates metal binding sites what results in the cleavage catalysis appearing for low ion concentrations. Indeed, our results showed similar cleavage pattern of M13mp18 plasmid in the presence of 0.5 mM, 0.75 and 1 mM Mn^2+^ pointing out that further increasing of the ion concentration does not improve the catalysis, which supports this model. Mg^2+^, on the other hand, exhibits lower binding constant and thus high concentration of the ion is necessary to saturate PA-Nter. However, the amount of Mg^2+^ ions added to saturate the protein highly depends on the co-purified ion level. Indeed, Stevaert *et al*. [28] also observed PA-Nter activity on the short substrates in the sample without addition of any ions, suggesting presence of co-purified ions. They reported the cleavage of molecular beacons with 1mM Mg^2+^ either 1mM Mn^2+^, however, when using ssDNA plasmid the cleavage was seen only with Mn^2+^. The authors argued that reaction of molecular beacon with Mg^2+^ was possible due to the intrinsic bending of substrate resulting in pre-reactive conformation of the scissile phosphodiester group. For that reason, we chose the sequence of 30-nt DNA hairpin having open conformation (Fig 3A) and showed that substrate bending cannot be the reason, since our substrate was cleaved by both Mn^2+^ and Mg^2+^ ions.

Wondering what the origin of co-purified ions is, we noticed that Crepin *et al*. [26], Dias *et al*. [11] and Datta *et al*. [27] all used the same PA-Nter purification protocol, finally concentrating the protein, what possibly had a positive contribution in additional ion removal. This may explain results of Datta *et al*. [27] who performed reaction for 2h in the buffer containing 2 mM DTT and observed PA-Nter activity in the presence of 1 mM Mn^2+^, but not with 1mM Mg^2+^ nor 1mM Mg^2+^ plus 0.1mM Mn^2+^. Optimal purification of PA-Nter suggests the higher concentration of Mg^2+^ is required to saturate metal binding sites. And thus, Dias *et al*. [11] used higher concentration of ions (1.5 mM) in 30 min reaction and reported cleavage of 81-nt partially structured RNA with Mg^2+^ either Mn^2+^ in buffer containing β-mercaptoethanol at pH 8. Similarly, Crepin *et al*. [26] observed cleavage catalysis of 51-nt U-rich RNA in the presence of 1mM Mg^2+^ either Mn^2+^, using the same buffer as Dias *et al*. and longer reaction time (80 min and 6h, respectively). Here, longer incubation time might facilitate the reaction with Mg^2+^.

To fully resolve the discrepancies between different experimental reports, more systematic studies are needed with an unambiguous and quantitative identification of metals in PA-Nter. Methods such as particle induced X-ray emission [40], are promising for this task, especially the scope of their applications is extending to non-crystalline samples or even whole cells. Metal binding issue is furthermore complicated by the fact, that PA-Nter from different virus strains are used in contradictory studies. Although the key active site residues (His41, Glu80, Asp108, Glu119, Lys134) are fully conserved among 13,000 sequences from influenza A, B and C [30], distinct full-length PA activity and even cleavage patterns for four influenza strains were observed [29]. It may suggest that the overall structure and tightness of ion binding is subject to strain-to-strain variations. Further studies on this topic would allow verifying this hypothesis.

### PA-Nter as a part of complete influenza polymerase complex

Although PA-Nter, as a small single-domain protein, is convenient to work with, it has to be remembered that it is only a part of heterotrimetic PB1-PA-PB2 polymerase complex. Recently, it has been shown for Influenza B polymerase-nuclear localization sequence (NLS) peptide complex, that for a capped RNA the nuclease activity was far higher (~100 times) than for the isolated nuclease domain [34]. The difference is most likely related to the presence of cap-binding PB2 domain. To ensure that PA-Nter exhibits similar ion preference as the complete polymerase, similar cleavage analysis should be performed on such version of protein as well as on purified ribonucleoprotein (RNP). RNP-mediated endonuclease reactions gave similar products as compared with PA-Nter, but different ionic conditions were used [27]. However, metal-depending cleavage studies with exclusive use of PA-Nter are still valuable, because in none of the existing structures metal ions were not present in other subunits than PA.

### Mg^2+^ as a more preeminent cofactor of PA-Nter

Mg^2+^ is one of the most abundant divalent cations in the living cells (~1 mM) [28] and it is outstanding in regard to solubility, redox stability, relative small size, rigid coordination geometry and hydration properties [22]. Therefore, from variable metals involved in the enzymatic catalysis, it was Mg^2+^, which was found to be the most frequently used by the nucleic acid enzymes. However, when the Mg-dependent enzyme is activated with other ions, the efficiency and/or substrate specificity might be reduced [32]. Indeed, Mn^2+^, which commonly replaces Mg^2+^ [41], exhibits less stringent coordination requirements, which lowers the substrate specificity and might rescue the deficient enzymes [22]. Moreover, Mn^2+^ is reported as a better Lewis acid and more efficiently than Mg^2+^ facilitates the formation of the attacking nucleophile [32]. Consequently, Mn^2+^ seems to be the preferred cofactor of PA-Nter, because of the reported maximum activity on the wide range of substrates [11, 27, 28, 42]. Although different authors suggested Mn^2+^ as a first-choice ion for PA-Nter, Xiao *et al*. [32] made an accurate observation that physiological concentration of Mn^2+^ (~1 μM), is not sufficient for the endonuclease activity, which required ~100 μM for optimal performance. It should be also mentioned that structural study of the endonuclease utilizes crystal soaking solutions containing high non-physiological concentration of divalent metals: 100 mM Mg^2+^ [13], 10 mM Mg^2+^ and 2,5 mM Mn^2+^ [11], 2 mM Mg^2+^ and 2 mM Mn^2+^ [31], 10 mM Mg^2+^ and 5 mM Mn^2+^ [30]. This issue has been already raised by Xiao *et al*. [32] and Zhao *et al*. [43]. Hence, it is noteworthy, that majority of the *in vitro* study of PA-Nter used artificial conditions, and therefore results might be difficult for unambiguous interpretation and translation into reality.

**Fig 6:**
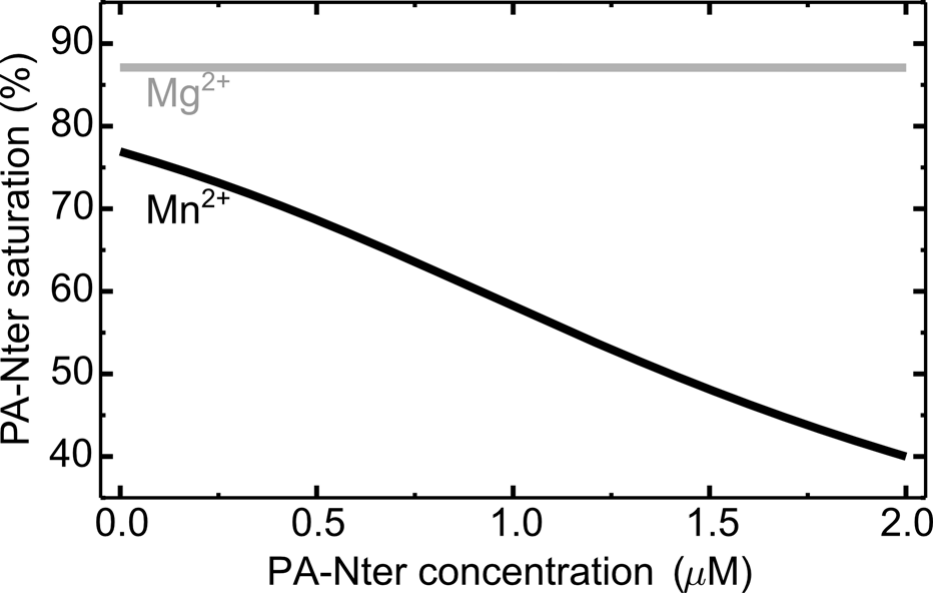
PA-Nter ion occupancy *in vitro*. The curves are plotted according to Eq 4, assuming 1 μM total concentration of Mn^2+^ and 1mM Mg^2+^.

To estimate PA-Nter occupancy in cells, we calculated it as *[PI]/P*_*tot*_ (see Material and Methods Eq 4), assuming total ion concentrations (Mn^2+^: 1μM, Mg^2+^: 1mM) for a range of PA-Nter concentrations (Fig 6). To our knowledge, the number of influenza polymerase molecules translated into proteins during replication cycle is not known precisely. Therefore we considered 2 μM concentration as the upper limit, which corresponds to ~5,000 protein copies in the volume of a typical endothelial cell nucleus of a 2 μm diameter. Already this variable range allowed the observation, that PA-Nter saturation with Mn^2+^ decreases rapidly, since the pool of free manganese ions is quickly consumed. Contrary, free Mg^2+^ ions are abundant and in spite of their higher *K*_*D*_ they saturate the protein at almost constant level of ~87%. This calculation shows, that *in vivo* both ions can be used as cofactors for low polymerase concentrations, however because of higher Mg^2+^ abundance, PA-Nter saturation with Mg^2+^ is higher.

## Supporting Information

S1 Fig Purification of PA-Nter. (TIF)

S2 Fig Digestion of ssDNA plasmid by PA-Nter not subjected to buffer exchange step. (TIF)

S3 Fig Possible secondary structures of single stranded oligonucleotides used in this study. (TIF)

S4 Fig Cleavage monitored in electrophoretic experiment. (TIF)

S5 Fig Control experiments for FCCS cleavage assay. (TIF)

S6 Fig Cleavage of unlabelled 30-nt DNA hairpin. (TIF)

S7 Fig Cleavage of ssDNA under reducing conditions. (TIF)

S1 Table Activity of PA-Nter in the presence of Mn^2+^ or Mg^2+^ reported by different authors. (DOC)
