# Correction: New Insight into Metal Ion-Driven Catalysis of Nucleic Acids by Influenza PA-Nter

**DOI:** 10.1371/journal.pone.0159121

**Published:** 2016-07-06

**Authors:** Daria Kotlarek, Remigiusz Worch

The following information is missing from the Financial Disclosure: Experiments were performed in the NanoFun laboratories co-financed by the ERDF Project POIG.02.02.00-00-025/09.
