# Supplementary figures and images for: New Insight into Metal Ion-Driven Catalysis of Nucleic Acids by Influenza PA-Nter
Source: PLoS One. 2016 Jun 14;11(6):e0156972. doi: 10.1371/journal.pone.0156972 (PMC4907508; doi:10.1371/journal.pone.0156972)

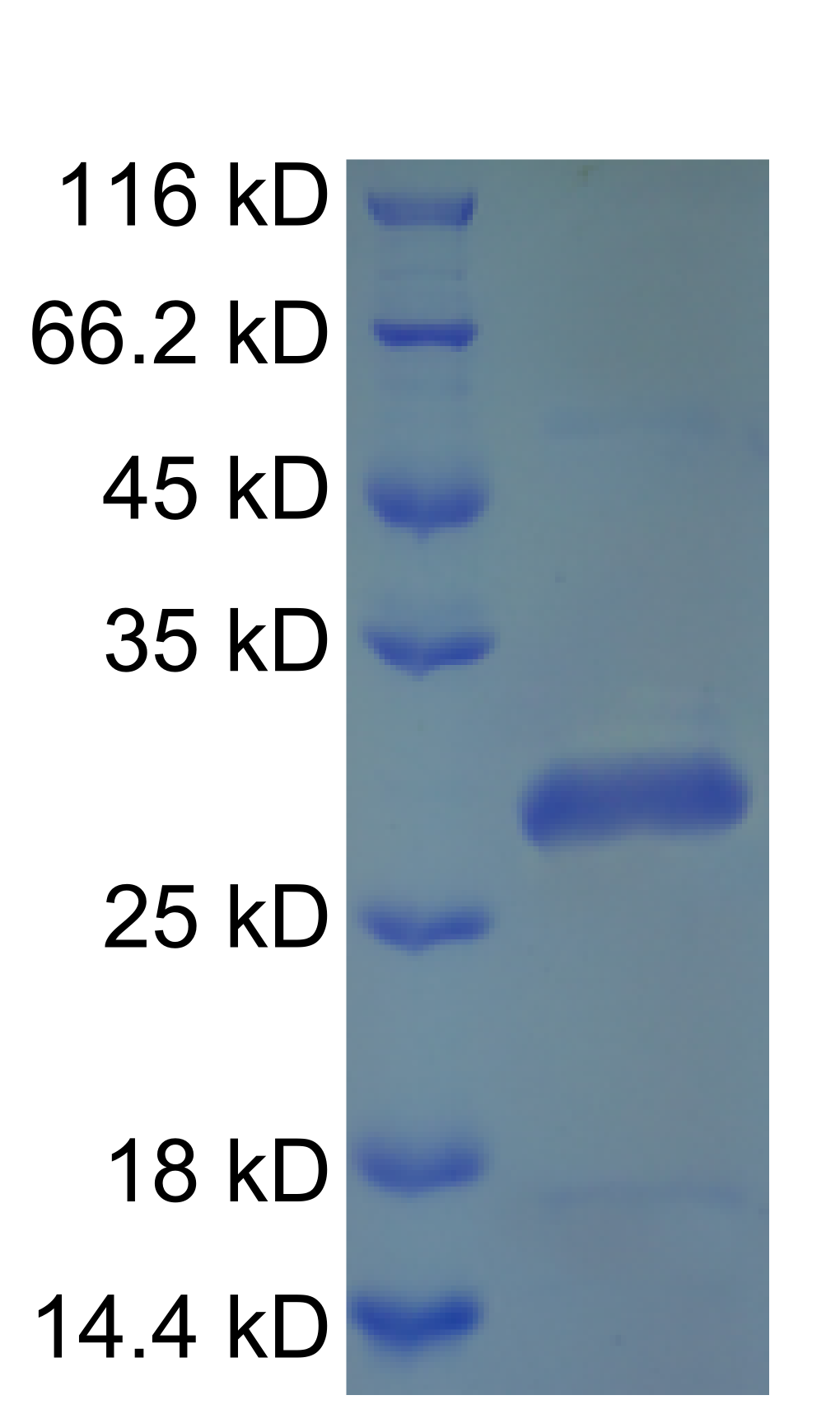

Supplement: S1 Fig — (TIF) [file pone.0156972.s001.tif]

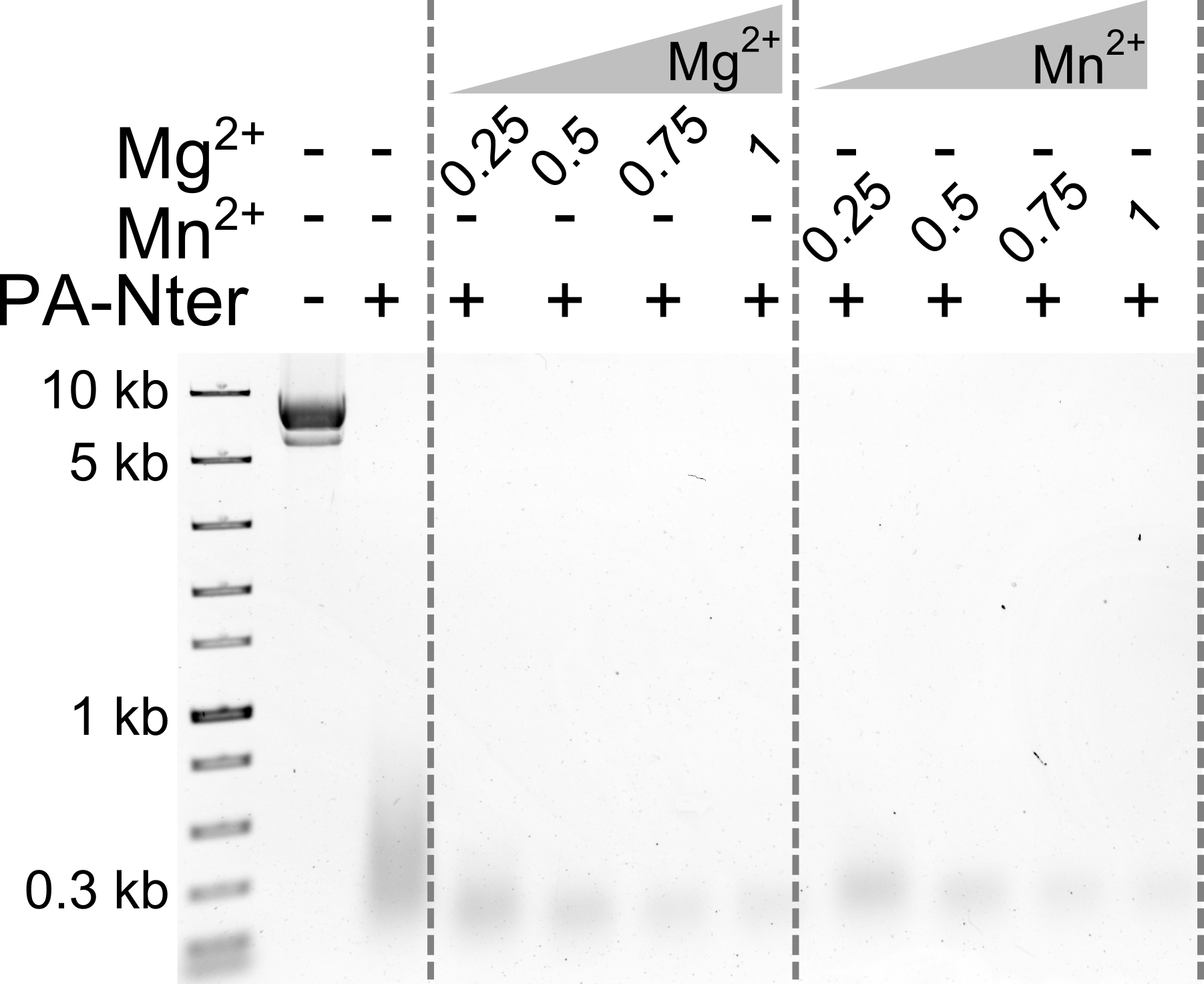

Supplement: S2 Fig — (TIF) [file pone.0156972.s002.tif]

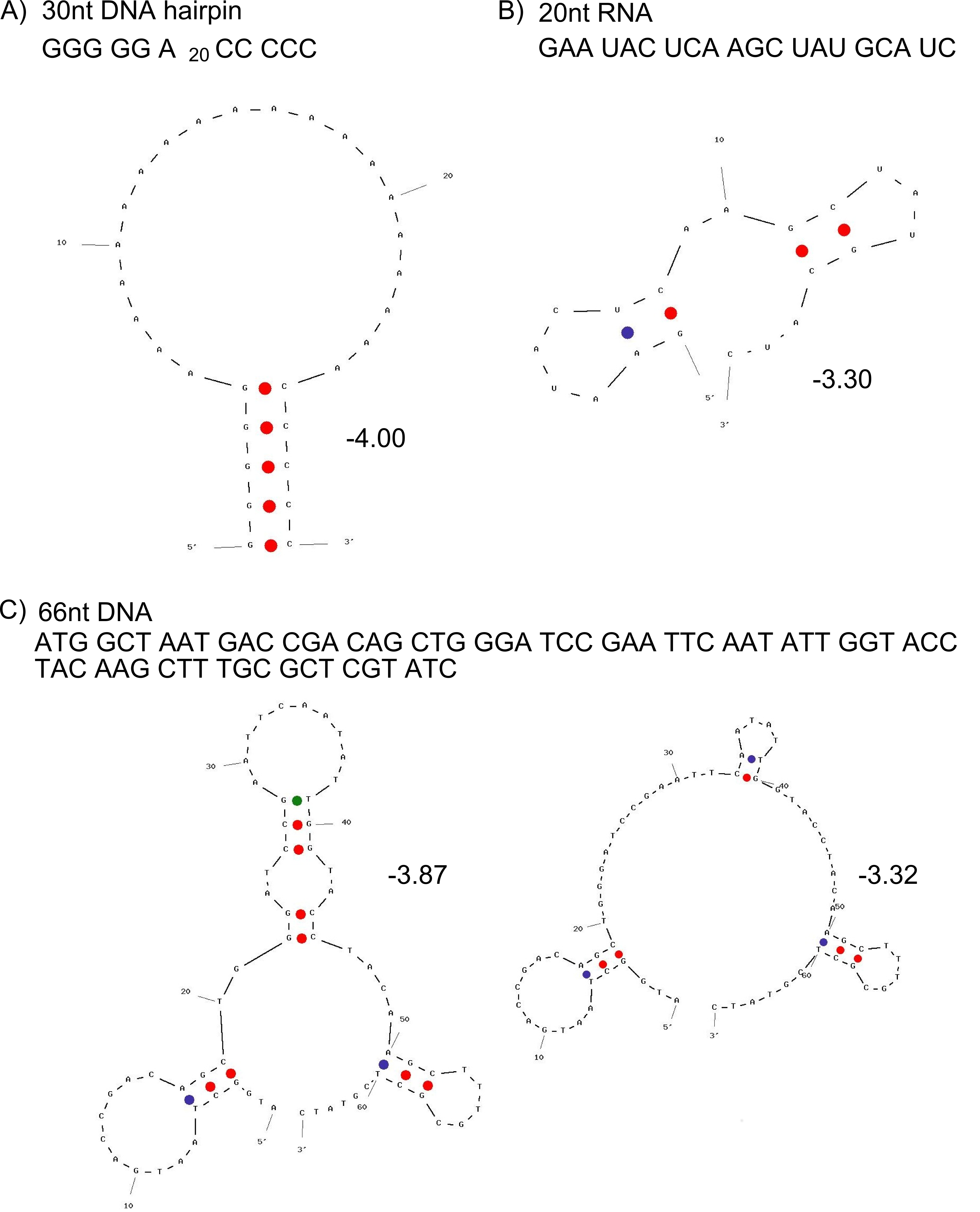

Supplement: S3 Fig — (TIF) [file pone.0156972.s003.tif]

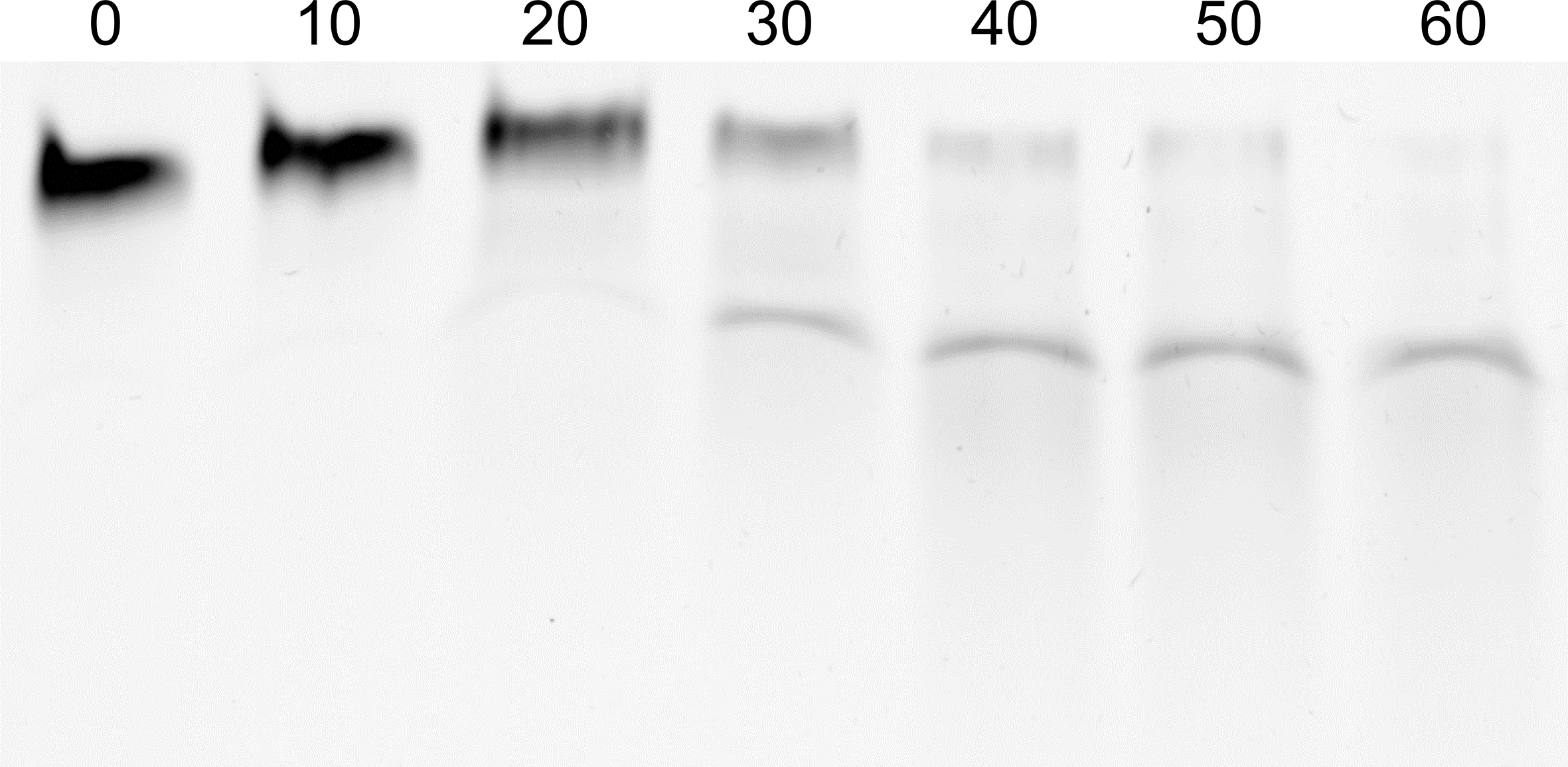

Supplement: S4 Fig — (TIF) [file pone.0156972.s004.tif]

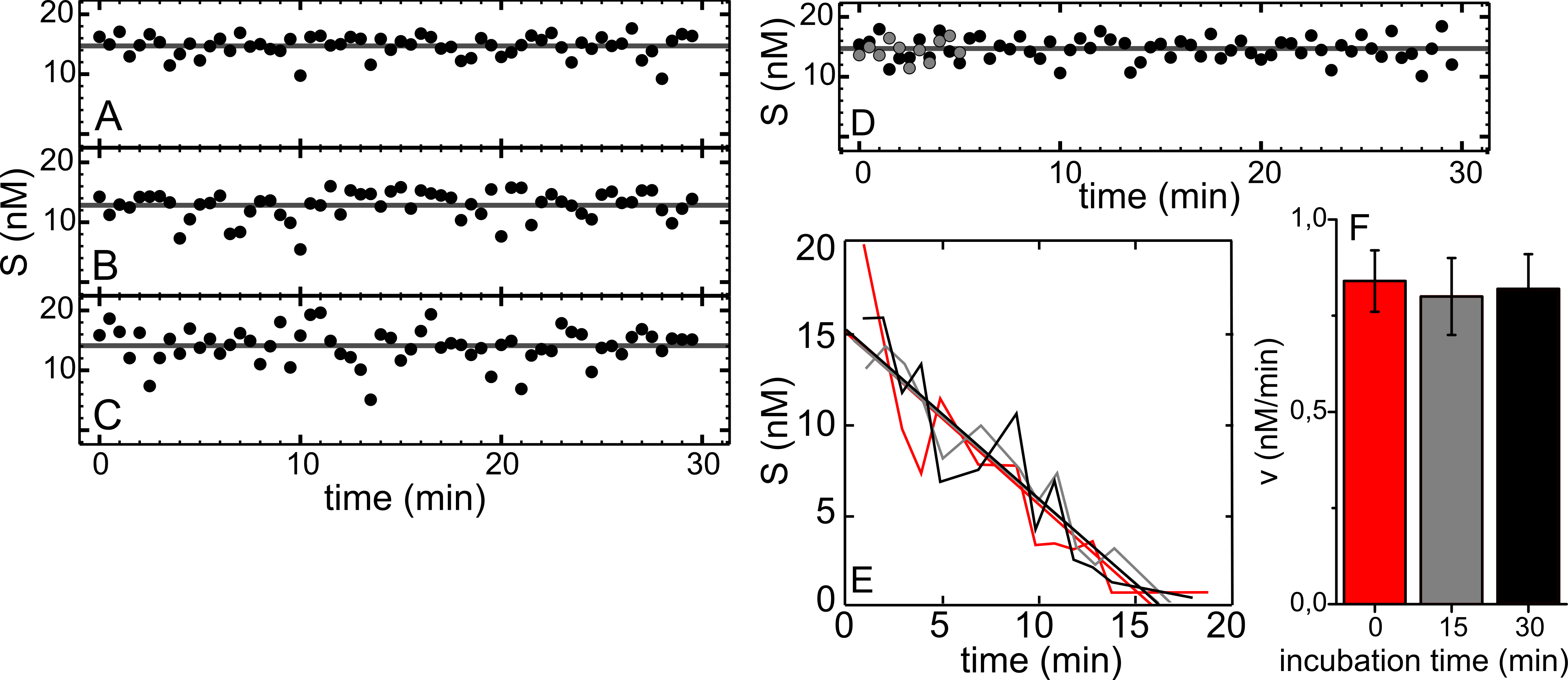

Supplement: S5 Fig — (TIF) [file pone.0156972.s005.tif]

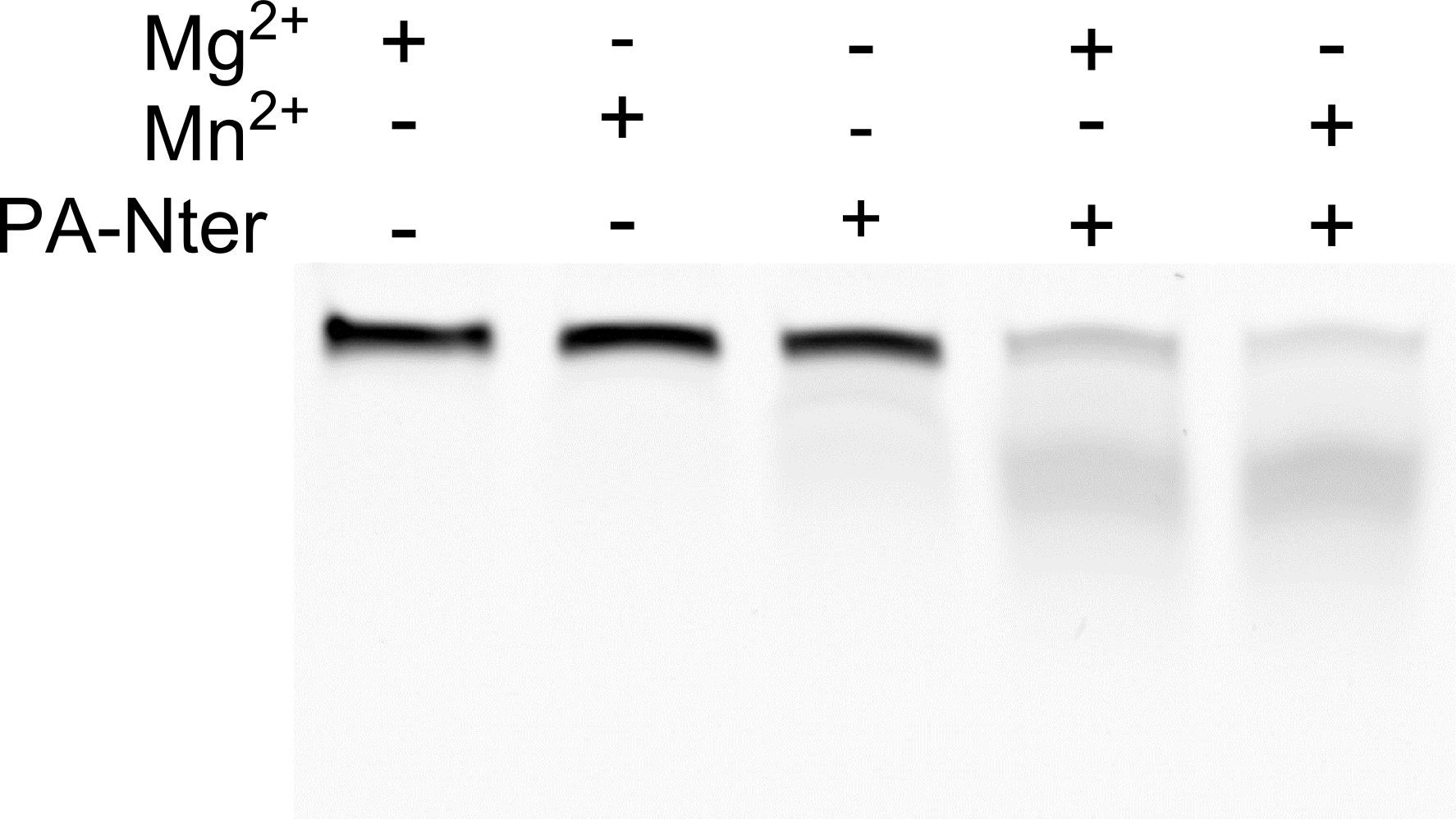

Supplement: S6 Fig — (TIF) [file pone.0156972.s006.tif]

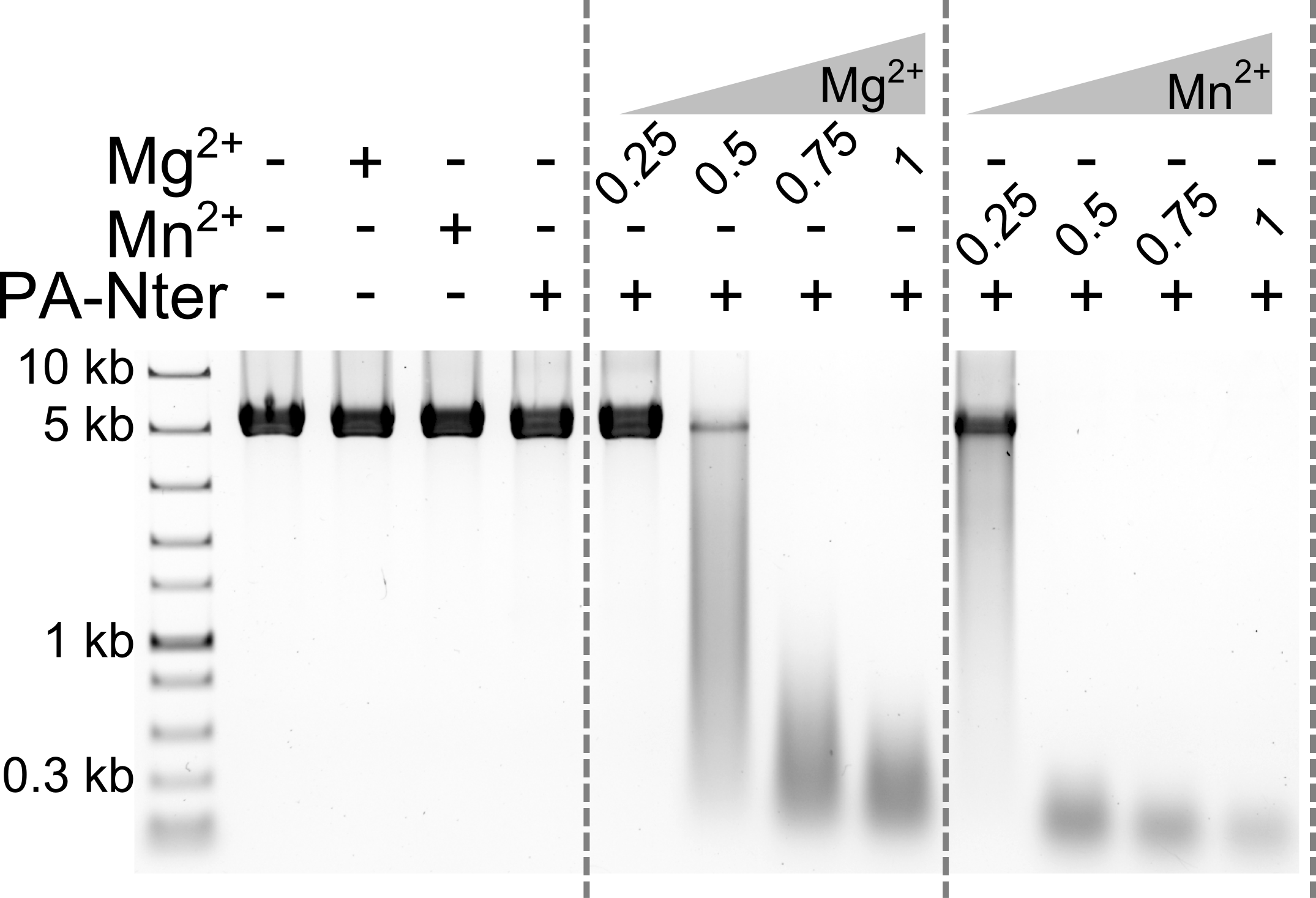

Supplement: S7 Fig — (TIF) [file pone.0156972.s007.tif]
