# Supplementary material for: New Insight into Metal Ion-Driven Catalysis of Nucleic Acids by Influenza PA-Nter
Source: PLoS One. 2016 Jun 14;11(6):e0156972. doi: 10.1371/journal.pone.0156972 (PMC4907508; doi:10.1371/journal.pone.0156972)
Supplement: S1 Table — (DOC) [file pone.0156972.s008.doc]

|  | Dias et al., 2009 [11] | Crépin et al., 2010 [26] | Datta et al., 2013 [27] | Stevaert et al., 2015 [28] |
| --- | --- | --- | --- | --- |
| Mn^2+^ | + | + | + | + |
| Mg^2+^ | +/- ^a^ | + | - | +/- ^b^ |

# Activity (+) or no activity (-) observed.

^a^ no cleavage at pH 7

^b^ no cleavage of 7.249 kb ssDNA plasmid
